# N-acetyl-L-cysteine reduces testis ROS in obese fathers but fails in protecting offspring from acquisition of epigenetic traits at *cyp19a1* and *IGF11/H19* ICR loci

**DOI:** 10.3389/fcell.2024.1450580

**Published:** 2024-10-18

**Authors:** Arianna Pastore, Nadia Badolati, Francesco Manfrevola, Serena Sagliocchi, Valentina Laurenzi, Giorgia Musto, Veronica Porreca, Melania Murolo, Teresa Chioccarelli, Roberto Ciampaglia, Valentina Vellecco, Mariarosaria Bucci, Monica Dentice, Gilda Cobellis, Mariano Stornaiuolo

**Affiliations:** ^1^ Department of Pharmacy, University of Naples “Federico II”, Naples, Italy; ^2^ Telethon Institute of Genetics and Medicine (TIGEM), Pozzuoli, Italy; ^3^ Department of Experimental Medicine, University della Campania “Luigi Vanvitelli”, Naples, Italy; ^4^ Department of Clinical Medicine and Surgery, Naples, Italy

**Keywords:** intergenerational inheritance, epigenetics, IGFII, H19, CYP19A1, DNA methylation, paternal obesity, diabetes

## Abstract

**Introduction:**

Paternal nutrition before conception has a marked impact on offspring’s risk of developing metabolic disorders during adulthood. Research on human cohorts and animal models has shown that paternal obesity alters sperm epigenetics (DNA methylation, protamine-to-histone replacement, and non-coding RNA content), leading to adverse health outcomes in the offspring. So far, the mechanistic events that translate paternal nutrition into sperm epigenetic changes remain unclear. High-fat diet (HFD)-driven paternal obesity increases gonadic Reactive Oxygen Species (ROS), which modulate enzymes involved in epigenetic modifications of DNA during spermatogenesis. Thus, the gonadic pool of ROS might be responsible for transducing paternal health status to the zygote through germ cells.

**Methods:**

The involvement of ROS in paternal intergenerational transmission was assessed by modulating the gonadic ROS content in male mice. Testicular oxidative stress induced by HFD was counterbalanced by N-acetylcysteine (NAC), an antioxidant precursor of GSH. The sires were divided into four feeding groups: i) control diet; ii) HFD; iii) control diet in the presence of NAC; and iv) HFD in the presence of NAC. After 8 weeks, males were mated with females that were fed a control diet. Antioxidant treatment was then evaluated in terms of preventing the HFD-induced transmission of dysmetabolic traits from obese fathers to their offspring. The offspring were weaned onto a regular control diet until week 16 and then underwent metabolic evaluation. The methylation status of the genomic region *IGFII/H19* and *cyp19a1* in the offspring gDNA was also assessed using Sanger sequencing and methylation-dependent qPCR.

**Results:**

Supplementation with NAC protected sires from HFD-induced weight gain, hyperinsulinemia, and glucose intolerance. NAC reduced oxidative stress in the gonads of obese fathers and improved sperm viability. However, NAC did not prevent the transmission of epigenetic modifications from father to offspring. Male offspring of HFD-fed fathers, regardless of NAC treatment, exhibited hyperinsulinemia, glucose intolerance, and hypoandrogenism. Additionally, they showed altered methylation at the epigenetically controlled loci *IGFII/H19* and *cy19a1.*

**Conclusion:**

Although NAC supplementation improved the health status and sperm quality of HFD-fed male mice, it did not prevent the epigenetic transmission of metabolic disorders to their offspring. Different NAC dosages and antioxidants other than NAC might represent alternatives to stop the intergenerational transmission of paternal dysmetabolic traits.

## 1 Introduction

The nutritional status of parents, both mothers and fathers, has a profound impact on the risk of dysmetabolism in offspring (Sharma and Rando, 2017; Power et al., 2003; Le Blévec et al., 2020), a risk that extends to several generations. Data from the Hunger-Winter cohort, individuals born during the Dutch famine in 1944, show that maternal undernutrition during gestation increases children’s susceptibility to diseases in later life. Indeed, adverse intrauterine environments, such as those affected by maternal starvation or obesity, increase the risk of type 2 diabetes and obesity in children (Lumey et al., 2007). In the Överkalix cohort, paternal grandfathers exposed to an excess of food in their childhood had an increased chance of their grandchildren presenting with increased BMI, waist circumference, and fat mass (Sharma and Rando, 2017; Pembrey et al., 2006).

So far, animal models have shown that a variety of paternal lifestyle factors, including obesogenic diets, are linked to adverse outcomes in offspring and persist for several generations (Gong et al., 2021; Oshio et al., 2020; Carone et al., 2010; Ng et al., 2010; Huypens et al., 2016; Watkins et al., 2018; Fullston et al., 2013). First, paternal HFD affects the sperm production process itself (Su et al., 2022). Obesity can lead to decreased testosterone levels, reduced sperm count, and sperm DNA fragmentation (Fan et al., 2015; Schagdarsurengin and Steger, 2016). Moreover, paternal obesity promotes gonadal oxidative stress, increases Reactive Oxygen Species (ROS), and causes chronic inflammation, both of which can lead to compromised sperm function and quality, further hindering male reproductive capabilities (Sanchez-Garrido et al., 2018; Aitken and Drevet, 2020). Preconceptional paternal HFD can induce changes in the sperm epigenome, including alterations in spermatocyte DNA methylation patterns and impairment of histone-to-protamine replacement. Despite the existence of maternal demethylases able to recognize protamine-bound paternal genome and demethylate it, several paternal genetic loci do not condensate around protamines and remain associated to histones, which protect paternal genes from maternal demethylation program (Bao and Bedford, 2016). Preconceptional paternal HFD also leads to improper histone acetylation (Terashima et al., 2015) and the transmission of small non-coding RNAs (Swanson et al., 2020; Perez and Ben, 2019; Chen et al., 2016). These alterations can influence gene regulation in zygotes and developing embryos (Swanson et al., 2020; Galan, Krykbaeva, and Rando, 2020).

To date, the molecules that transduce signals and inform the sperm epigenome of individuals with paternal obesity remain largely unknown. During spermatogenesis, ROS modulate DNA methyltransferase, histone deacetylase, and acetylases, as well as RNA polymerase activity (Kietzmann et al., 2017). Thus, the ROS pool in the testes might deliver information on fathers’ nutritional status to the epigenome of sperm and zygotes (Yoshida et al., 2020).

At physiological levels, ROS are essential for sperm maturation and act as signaling molecules for the proper progression of spermatogenesis. ROS participate in intracellular signaling cascades and regulate proliferation and differentiation of spermatogonia into mature spermatozoa (Agarwal et al., 2016; Aitken and Drevet, 2020), acrosome reaction, and a plethora of sperm plasma membrane functions necessary for successful egg fertilization. Physiological levels of peroxides, the primary radical species found in the epididymal fluid, semen, and sperm cells, play a crucial role for entry of sperm cells located in the epididymis into senescence. ROS are involved in sperm capacitation, a process that enhances sperm plasma membrane fluidity through oxidation and sterol extrusion, promoting hyperactivated motility of the sperm flagellum, binding of the sperm to the zona pellucida for fertilization.

Excess ROS can have detrimental effects on sperm cells and lead to oxidative stress, oxidative DNA damage, lipid peroxidation, and apoptosis, thereby affecting male fertility (Tremellen, 2008). Recent evidence suggests that epigenetic alterations in sperm cells might be linked to sperm oxidative stress. Indeed, DNA methyltransferases (DNMTs), the enzymes responsible for adding methyl groups to DNA, are modulated by ROS (Darbandi et al., 2019a; Darbandi et al., 2019b). The inhibition of DNMTs under oxidative conditions may consequently result in global sperm DNA hypomethylation and affect gene expression in zygotes and in the first stage of embryo development.

The hypothesis that gonadal ROS imprint epigenetic information into sperm gDNA and transduce deleterious paternal information to the zygote suggests that re-establishing the redox balance in gonadal cells could interrupt the paternal transmission of pro-dysmetabolic epigenetic traits. Antioxidants could counteract the negative effects exerted by unhealthy lifestyle habits (HFD, smoking, and alcohol abuse) on paternal health, improving systemic factors, reducing inflammation, and creating a more favorable environment for healthy sperm production (Aitken and Drevet, 2020). Moreover, antioxidants could neutralize excess gonadal ROS and reduce aberrant epigenetic modifications in sperm cells, thereby reducing the transmission of deleterious epigenetic information. Ascorbic acid, α-tocopherol, polyunsaturated fatty acids, trace elements (mainly zinc and selenium), carnitine, N-acetylcysteine (NAC), Coenzyme Q10, and folates have been evaluated for their ability to improve male gametogenesis, modulate germ cell epigenetics, and potentially restore the metabolic health of offspring affected by early-life stressors (Pascoal et al., 2022).

We have recently shown that, in a murine model, pre-conception paternal HFD affects the epigenetic state of offspring *cyp19a1*, a gene coding for Aromatase, the cytochrome converting testosterone into 17-β estradiol (both potent hormonal mediators of embryo development and metabolism) (Pastore et al., 2024) and of the imprinted control region allocated in the *IgFII-H19* region. By affecting the methylation status of these regions, the paternal diet coordinates androgen metabolism in the progeny, promoting testosterone reduction, hypoandrogenism, growth retardation, and diabetes in male pups.

To verify whether treatment with an antioxidant could interfere with deleterious paternal epigenetic transmission, we fed male sires an HFD in the absence or presence of NAC, an antioxidant used by idiopathic infertile males (Jannatifar et al., 2019). We showed that NAC protects sires from the systemic (obesity, glucose intolerance, and insulin resistance) and gonad-specific (epididymal adiposity, epididymal, and gonadal oxidative stress) effects of the obesogenic diet, making their health status and morphometric parameters comparable to those of sires fed the control diet. Notwithstanding, despite the preservation of paternal health, NAC does not hamper the epigenetic transmission of dysmetabolic traits to the offspring, which is similar to those born from NAC-untreated fathers presenting paternally imprinted hypoandrogenism, growth retardation, and diabetes.

## 2 Materials and methods

### 2.1 Animal procedures

The animal procedures were approved by the Institutional Animal Care and Use Committee (IACUC) under protocol No. 846/2020-PR. All animal experiments complied with the ARRIVE guidelines (Pastore et al., 2024), the United Kingdom. Animals (Scientific Procedures) Act of 1986, and the EU Directive 2010/63/EU for animal experiments. C57BL/6J Ola mice (Envigo, Europe) were housed in groups of five per cage under controlled conditions with a 12:12 h light-dark cycle. Following a 2 week acclimatization period, five-week-old F0 male mice were divided into four groups based on similar average body weights. The control group (F0-CON, n = 30) received a standard diet (Teklad Rodent Diet, # TD-8604: 3; Envigo, Europe), while the HFD (F0-HFD, n = 30) group was fed HFD (Teklad Rodent Diet, # TD-06414: 5.1 kcal/g, 60.3% fat, 18.3% protein, and 21.4% carbohydrates; Envigo, Europe). The NAC control group (F0-CON-NAC, n = 10) received a standard diet and *ad libitum* access to 2 g/L NAC diluted in drinking water (Ma, Gao, and Liu, 2016), while the NAC HFD (F0-HFD-NAC, n = 10) group received a HFD and 2 g/L NAC diluted in drinking water. After 8 weeks on their respective diets, F0 males from each group were mated with 13-week-old C57BL/6J Ola females (n = 160) consuming the control diet. These nulliparous, non-diabetic females exhibited no differences in body weight, fasting blood glucose, or plasma insulin concentration 1 week before mating. During mating, one male was paired with two females with *ad libitum* access to food and plain water. Successful mating was confirmed by the presence of vaginal plugs. F0 males were euthanized after an overnight fast 1 week after mating. F0 mice were euthanized using 100% carbon dioxide, which was infused into the cage at 50% VDR/min by using a flow meter.

### 2.2 Mating and offspring

The mating and offspring details for F0-CON and F0-HFD males are described (Pastore et al., 2024). Of the 20 mated F0-CON-NAC and F0-HFD-NAC pairs, all resulted in successful pregnancies. Throughout pregnancy and lactation, the females were fed a CON diet. Litter size was reduced to five pups by randomly removing excess pups to standardize postnatal nutrition. Following weaning, one male and one female pup from each litter were randomly chosen. The F1 generation from F0-HFD-NAC was denoted as F1(F0-HFD-NAC) (n = 10 per sex, each from a different litter), and the generation from F0-CON was labeled as F1(F0-CON-NAC) (n = 10 per sex, each from a different litter). Irrespective of the paternal lineage, male and female offspring were provided with a control diet (14% kcal fat) from weaning until week 13, at which point all animals underwent metabolic testing, biochemical analyses, and post-mortem necropsy. F1 offspring were euthanized at 16 weeks of age, and their tissues and blood snap-frozen in liquid nitrogen and subsequently stored at −80°C for further analysis. F1 mice were euthanized with 100% carbon dioxide.

Blood and tissue collection occurred between 9 a.m. and 1 p.m., with groups randomized across this period to accommodate variations in the fasting state and diurnal hormone secretion.

### 2.3 Oral glucose tolerance test (OGTT)

OGTT was conducted on 80 F0 sires and 212 F1 offspring (divided per group and per sex) 2 weeks before euthanasia. Prior to the OGTT, the animals fasted for 6 h. Each animal received a dose of glucose at 2 g per kilogram of body weight (20% w/v glucose) *via* gavage, and blood glucose levels were measured at 0, 15, 30, 60, 90, and 120 min using a Contour Next Glucose meter (Bayer, Basel, Switzerland). Insulin levels were determined using an ELISA kit (Insulin Mouse ELISA Kit, # ab277390; Invitrogen, Thermo Fisher Scientific, United States). Plasma concentrations of total cholesterol, high-density lipoprotein cholesterol (HDL), low-density lipoprotein cholesterol (LDL), triglycerides, aspartate aminotransferase (AST), and alanine aminotransferase (ALT) were measured using commercially available kits (Diacron International (Grosseto, Italy) and analyzed using a Diacron International analyzer. Gross necropsy and organ weight assessments were conducted as described previously (Pastore et al., 2024).

### 2.4 Sperm parameters

Each animal’s testes were promptly removed and stored at −80°C for molecular analysis or fixed in Bouin’s solution for histological examination. The epididymis was dissected into the caput, corpus, and cauda, and sperms were collected immediately. Briefly, each region was immersed in 3 mL of PBS (pH 7.6) and gently cut to release the sperm. The collected sperm samples were filtered through cheesecloth for further functional and biochemical analyses. Caput, corpus, and cauda sperms were utilized to evaluate total ROS, whereas cauda sperms were specifically utilized to assess sperm quality parameters such as count, motility, and viability as well as mitochondrial ROS and Glutathione (GSH) levels, as described below.

For caudal sperm quality assessments, sperm number, motility, and viability were evaluated under a light microscope using a hemocytometer. Sperm viability was determined using trypan blue dye, and sperm motility was assessed visually. At least 100 sperm cells were counted for each analysis. To measure oxidative stress in sperm, total ROS, mitochondrial ROS, and total reduced glutathione (GSH) levels were quantified using specific probes (Mitotracker CMX-ROS, #M7512, dichloro-fluorescein diacetate (CM-H2DCFDA), #C6827, and TAMRA-iodoacetamide (TMR-IAA), #T6006; Invitrogen, United States) dissolved in DMSO and incubated with sperm samples. The samples were fixed and analyzed using a fluorescence microscope.

### 2.5 Testosterone and estradiol measurement

Testosterone and 17-β estradiol levels in testicular tissue were quantified by homogenizing the testes in 70% methanol, extracting steroids in diethyl ether, and performing ELISA assays using commercial kits as previously described (Pastore et al., 2024). The standard curves provided by the manufacturer were used for quantification.

### 2.6 gDNA extraction and methylation analysis

Methylation-sensitive qPCR was performed as previously described (Pastore et al., 2024). Briefly GenomicDNA (gDNA) was extracted from (approximately 25–30 mg of) mouse pancreas and testis. 80 ng of each DNA sample were digested with 1 µL of HpaII (# ER0511, Thermo Fisher) or MspI (# ER0541, Thermo Fisher) in 1X EpiJET Buffer (#K1441, Thermo Fisher) and in a final reaction volume of 20 µL. The digestion was carried out overnight at 37°C to be then amplified by qPCR with the following cycling conditions: 95°C for 3 min followed by 40 cycles of [30 s of denaturation at 95°C, 30 s of annealing at 52°C; 30 s of amplification at 60°C] and the following primers: *CYP19A1 brain exon I:* Forward 5′-CTA​TCC​GGT​TTT​TAA​ACG​GC-3’; Reverse 5′-GGA​TCT​GCT​GGT​CAC​TTC​TA-3’; *IGFII/H19 ICR* Forward 5′GGA​ACC​GCC​AAC​AAG​AAA​GT-3’; Reverse 5′-GGT​CTT​TCC​ACT​CAC​AAC​GG-3’. The analysis of the methylation state of each target sequence was conducted by comparison of amplification Ct of digested samples with undigested control samples following the comparative 2^−ΔΔCT^ method.

*Sequencing of Bisulfite Converted gDNAs.* Bisulfite conversion was performed as described previously (Pastore et al., 2024). Two micrograms of extracted gDNA were bisulfite-converted using the EpiTect Bisulfite Kit (# 59104, Qiagen). Upon bisulfite conversion, regions of interest were amplified by qPCR with the following primers: for *cy19a1 brain exon I* forward primers was 5′-CTATCYGGTTTTTAAACGGC-3′ while reverse primer was 5′-GGA​TCT​GCT​GGT​CAC​TTC​TA-3’; for *IGFII/H19 ICR* forward primer was 5′-TTTATTTATAAYGGTTTTTGTGTTTTTT-3’; Reverse was 5′-AAA​CCC​CTA​ACT​AAC​TAA​TTT​ATA​ACA​AAT​AA-3.’ The PCR products were gel-purified and cloned into the TOPO-TA cloning vector system (TOPO TA Cloning Kit for Sequencing, Thermo Fisher Scientific, United States, #K4575J10). After transformation and plating on LB-Agar plates, 100 bacterial colonies were selected for plasmid purification. Purified plasmids were sequenced according to Sanger sequencing by Eurofins Scientific (Luxembourg) to determine the number and position of C and U and to depict the methylation patterns of the loci.

*Statistical analysis*. Statistical analyses were performed using Prism 6 software (GraphPad Software). Comparisons between groups were performed using a one-way analysis of variance (ANOVA), correcting for multiple comparisons using the Tukey method. Statistical significance was expressed as a *p*-value. Differences with *p* values >0.05 were considered non-statistically significant and were not indicated.

## 3 Results

**FIGURE 1:**
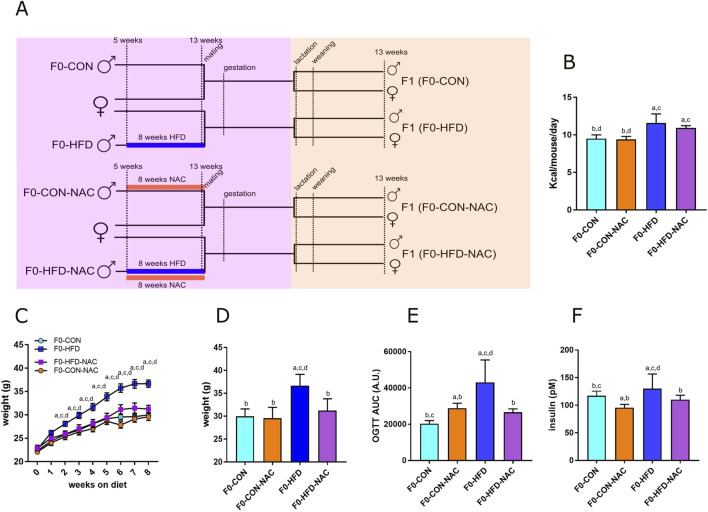
NAC protects F0-HFD-NAC males from HFD-induced acquisition of a pre-diabetic phenotype. **(A)** Feeding and breeding scheme used in this study. Five weeks old C57BL/6J males (F0) were fed either a control (CON) diet (F0-CON, n = 30), HFD diet (F0-HFD, n = 30), CON diet in the presence of NAC (F0-CON-NAC, n = 10), or HFD in the presence of NAC (F0-HFD-NAC, n = 10) for 8 weeks, to be then mated to inbred C57BL/6J females fed a regular CON diet. F1 from F0-CON (here named F1(F0-CON), n = 43 per sex, each from a different mother), from F0-HFD (here named F1(F0-HFD), n = 43 per sex, each from a different mother), F0-CON-NAC (here named F1(F0-CON-NAC), n = 20 per sex, each from a different mother) and F1 from F0-HFD-NAC (here named F1(F0-HFD-NAC), n = 20 per sex, each from a different mother) were fed CON diet from weaning to week 17 to then undergo metabolic testing, biochemical analyses and post-mortem necropsy. **(B–D)** Food consumed, body weight, weekly gain **(C)** and at necropsy **(D)** of sires belonging to the four different groups. **(E, F)** OGTT test **(E)** and fasting insulinemia **(F)** of sires from different groups. Data are presented as means and SD. Bars are labeled with *p* values: **(A)** (*p* < 0.05 vs. F0-CON); **(B)** (*p* < 0.05 vs. F0-HFD); **(C)** (*p* < 0.05 vs. F0-CON-NAC); **(D)** (*p* < 0.05 vs. F0-HFD-NAC).

We have already described and used a model of intergenerational paternal epigenetic transmission, where C57BL/6J inbred F0 males received an obesogenic stimulus (HFD, 60% of calories from fat) for 8 weeks before mating (Pastore et al., 2024). We have shown that in F0 sires, this stimulus promotes a prediabetic phenotype characterized by weight gain, visceral fat expansion, glucose intolerance, moderate hyperinsulinemia, and gonadal oxidative stress (increased ROS, decreased GSH content, and reduced sperm viability) (Pastore et al., 2024). Despite HFD has been shown to reduce sperm number (Sanchez-Garrido et al., 2018), our feeding scheme did not affect sperm number of HFD F0 males. Moreover, although the length of the treatment was long enough to affect the entire cycle of spermatogenesis (a process that takes 35 days in mice), the obesogenic stimulus did not affect male fertility (Pastore et al., 2024) (Figure 1A). Here, we tested the protective effect of chronic administration of NAC in the drinking water of HFD-fed mice. Owing to its strong antioxidant potential, NAC has been extensively studied for its potential to reestablish the redox balance in the testis and is used for the treatment of male idiopathic infertility (Dimitriadis et al., 2023; Su et al., 2022). Moreover, NAC protected mice from diet-induced obesity and metabolic disorders. Ma *et al.* showed that administering NAC in the drinking water of C57BL/6 mice protected male mice from HFD-induced metabolic disorders (Ma et al., 2016).

### 3.1 NAC protects F0 males from HFD-induced dysmetabolism and reduces gonadic ROS levels

The four groups of sires described in this manuscript were all part of the same animal cohort. The dysmetabolic parameters resulting from the HFD obesogenic stimulus in F0-HFD sires have been previously described (Pastore et al., 2024) and are reported here as reference parameters and used for statistical comparison. This manuscript focuses on the NAC receiving groups and their progeny. As shown in Table 1 and Figure 1, after 8 weeks of HFD, compared to F0 mice fed CON (F0-CON group, n = 30), F0 mice fed HFD (F0-HFD group, n = 30) showed increased body weight at sacrifice. Interestingly, F0 mice fed HFD and drinking NAC (F0-HFD-NAC group, n = 10) present body weight comparable to F0-CON and F0 mice fed CON and drinking NAC (F0-CON-NAC group, n = 10). NAC protects as well HFD fed mice from acquiring moderate hyperinsulinemia, fasting hyperglycemia and improves glucose sensitivity during OGTT test. WB analysis performed on liver homogenates reveal for each of the group comparable intracellular levels of phosphorylated AKT and AMPK two intracellular sensors of dysmetabolism (Pastore et al., 2024).

**TABLE 1:** Morphometric analysis (whole body, main organs and reproductive tract), hematochemical analysis, sperm analysis, sperm ROS staining (staining with DCF-DA, luminol or Mito Tracker CMX-ROS), GSH (quantitation by TMR-IAA staining) and fertility rate assessment performed on adult F0-CON, F0-HFD, F0-CON-NAC and F0-HFD-NAC.

	*F0-CON*	*F0-HFD*	*F0-CON-NAC*	*F0-HFD-NAC*
*Fathers (F0 generation)*	n = 30	n = 30	n = 10	n = 10
*Body Weight at sacrifice (g)*	30.0 ± 1.1^a^	36.7 ± 1.4^b^ ^,^ ^c^ ^,^ ^d^	29.6 ± 2.3^a^	31.2 ± 2.5^a^
*Energy Intake (Kcal/mice/day)*	9.5 ± 0.5^a^ ^,^ ^d^	11.6 ± 1.2^b^ ^,^ ^c^	9.4 ± 0.4^a^ ^,^ ^d^	10.9 ± 0.3^b^ ^,^ ^c^
*Organ weight*				
*Liver (g)*	1.23 ± 0.28	1.33 ± 0.22	1.27 ± 0.06	1.25 ± 0.05
*Testicle (g)*	0.053 ± 0.028	0.051 ± 0.022	0.054 ± 0.005	0.052 ± 0.004
*Prostate (g)*	0.060 ± 0.044	0.057 ± 0.033	0.058 ± 0.009	0.056 ± 0.005
*Seminal vesicles (g)*	0.24 ± 0.09^a^	0.26 ± 0.02^b^ ^,^ ^c^ ^,^ ^d^	0.23 ± 0.05^a^	0.23 ± 0.07^a^
*Abdominal fat (g/cm* ^*3*^ *)*	0.11 ± 0.02^a^	0.22 ± 0.03^b^ ^,^ ^c^ ^,^ ^d^	0.10 ± 0.02^a^	0.13 ± 0.05^a^
*Epidydimal Fat (g)*				
* Distal*	0.123 ± 0.050^a^	0.294 ± 0.105^b^ ^,^ ^c^ ^,^ ^d^	0.126 ± 0.007^a^	0.143 ± 0.010^a^
* Central*	0.062 ± 0.028^a^	0.179 ± 0.105^b^ ^,^ ^c^ ^,^ ^d^	0.073 ± 0.004^a^	0.082 ± 0.012^a^
* Proximal*	0.036 ± 0.022^a^	0.102 ± 0.061^b^ ^,^ ^c^ ^,^ ^d^	0.033 ± 0.006^a^	0.052 ± 0.008^a^
* Caudal*	0.007 ± 0.006^a^	0.012 ± 0.006^b^ ^,^ ^c^ ^,^ ^d^	0.006 ± 0.001^a^	0.008 ± 0.001^a^
*Clinical Biochemistry*				
*Glucose (mg/dL)*	83 ± 4^b^ ^,^ ^a^ ^,^ ^d^	106 ± 7^b^ ^,^ ^c^ ^,^ ^d^	81 ± 5^a^ ^,^ ^d^	96 ± 6^b^ ^,^ ^a^ ^,^ ^c^
*Insulin (pM)*	117.3 ± 8.18^a^ ^,^ ^c^	130.1 ± 26.4^b^ ^,^ ^c^ ^,^ ^d^	95 ± 6^b^ ^,^ ^a^	110 ± 8^a^
*OGTT (AUC)*	20,276 ± 1752^a^ ^,^ ^c^	43,095 ± 12,335^b^ ^,^ ^c^ ^,^ ^d^	28,872 ± 2,727^b^ ^,^ ^a^	26,578 ± 1886^a^
*Cholesterol Total (mg/dL)*	92.4 ± 12.7^a^	110.39 ± 23.5^b^ ^,^ ^c^ ^,^ ^d^	88.4 ± 14.0^a^	83.4 ± 14.9^a^
*Triglycerides (mg/dL)*	139.2 ± 15.8^a^ ^,^ ^c^	160.9 ± 20.2^b^ ^,^ ^c^ ^,^ ^d^	107.8 ± 7.8^b^ ^,^ ^d^	126.4 ± 34.6^a^
*HDL (mg/dL)*	43.2 ± 6.1^a^ ^,^ ^c^ ^,^ ^d^	39.6 ± 3.7^b^ ^,^ ^c^ ^,^ ^d^	57.3 ± 5.8^a^ ^a^ ^,^ ^d^	47.4 ± 5.8^a^ ^,^ ^c^
*LDL (mg/dL)*	11.9 ± 0.9^a^ ^,^ ^c^ ^,^ ^d^	8.9 ± 0.8^a,c^	10.4 ± 0.8^b^ ^,^ ^a^	9.6 ± 1.1^b^
*ALT (U/L)*	31.3 ± 2.1^a^ ^,^ ^c^ ^,^ ^d^	33.2 ± 2.0^b^ ^,^ ^c^ ^,^ ^d^	27.0 ± 2.2	25.4 ± 1.7
*AST (U/L)*	51.2 ± 5.0	53.3 ± 6.0	57.1 ± 5.7	55.2 ± 5.3
*Sperm parameters*				
*Concentration (x 10* ^*6*^ */mL)*	9.7 ± 1.5	10.1 ± 2.1	9.2 ± 0.4	10.1 ± 0.6
*Motility (caudal, %)*	72.8 ± 3.7	72.2 ± 6.5	73.6 ± 2.8	73.0 ± 4.9
*Viability (%)*	57.1 ± 1.3^a^	41.0 ± 3.8^b^ ^,^ ^c^ ^,^ ^d^	56.0 ± 1.6^a^	57.7 ± 1.0^a^
*ROS (DCF-DA) (% positive cells)*				
*Total spermatozoa*	13 ± 2^a^	22 ± 2^b^ ^,^ ^c^ ^,^ ^d^	13 ± 3^a^	14 ± 3^a^
*Caput spermatozoa*	24 ± 2^a^	57 ± 2^b^ ^,^ ^c^ ^,^ ^d^	26 ± 3^a^	25 ± 3^a^
*Corpus spermatozoa*	17 ± 2^a^	28 ± 3^b^ ^,^ ^c^ ^,^ ^d^	16 ± 1^a^	18 ± 2^a^
*Cauda spermatozoa*	11 ± 2^a^	19 ± 2^b^ ^,^ ^c^ ^,^ ^d^	10 ± 3^a^	11 ± 3^a^
*ROS (Luminol reaction) (U/mg of protein)*				
*Cauda spermatozoa*	0.8 ± 0.1^a^	3.3 ± 0.3^b^ ^,^ ^c^ ^,^ ^d^	0.8 ± 0.1^a^	0.9 ± 0.2^a^
*Epididymis*	0.5 ± 0.1^a^	5.3 ± 0.3^b^ ^,^ ^c^ ^,^ ^d^	0.6 ± 0.1^a^	0.6 ± 0.1^a^
*Testicle*	0.1 ± 0.1	0.1 ± 0.1	0.1 ± 0.1	0.1 ± 0.1
*Prostate*	0.1 ± 0.1	0.1 ± 0.1	0.1 ± 0.1	0.1 ± 0.1
*Seminal vesicles*	0.1 ± 0.1	0.1 ± 0.1	0.1 ± 0.1	0.1 ± 0.1
*Mitochondrial ROS (Mito Tracker CMX-ROS, FI)*				
*Caudal spermatozoa*	7,937 ± 1,092	8,843 ± 1,320	8,237 ± 995	8,169 ± 1,100
*Glutathione (Fluorescence-TMR-IAA)*				
*Caudal spermatozoa*	674 ± 49^c^ ^,^ ^d^	683 ± 49^c^ ^,^ ^d^	810 ± 15^b^ ^,^ ^a^	779 ± 14^b^ ^,^ ^a^
*Fertility rate (%)*	100	100	100	100
*Average number of pups*	6 ± 0.2	5.8 ± 0.2	5.7 ± 0.3	5.9 ± 0.3

Results are reported as mean ± SD., Statistically significant difference and values *p* values <0.05 are indicated with letters.

^a^ (*p* < 0.05 vs. F0-HFD).

^b^ (*p* < 0.05 vs. F0-CON).

^c^ (*p* < 0.05 vs. F0-CON-NAC).

^d^ (*p* < 0.05 vs. F0-HFD-NAC).

### 3.2 NAC reduces gonadic ROS levels protects F0 males from gonadic oxidative stress

**FIGURE 2:**
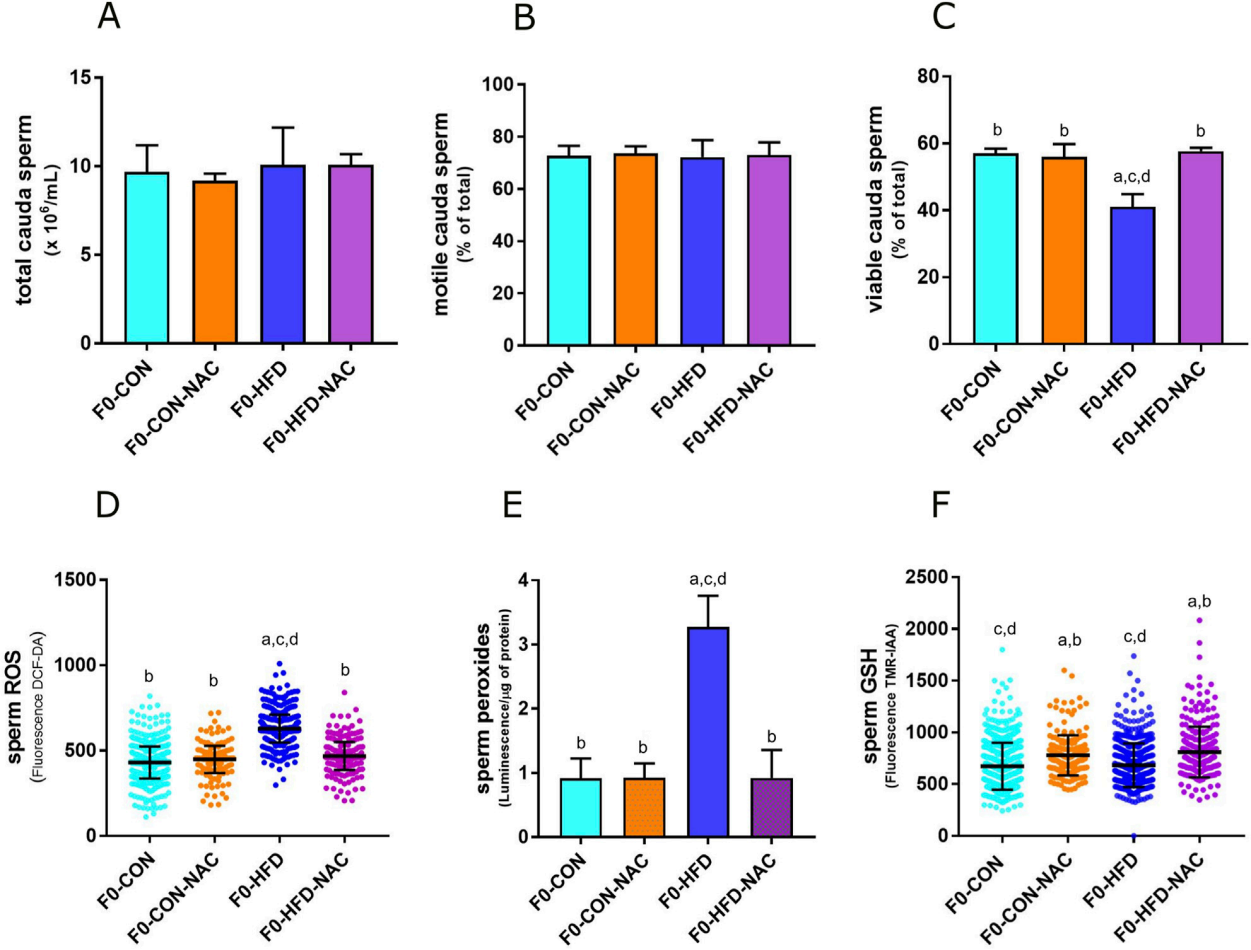
NAC counterbalances oxidative stress in gonads of F0-HFD-NAC males and re-establishes sperm viability. **(A–C)** Concentration **(A)**, motility **(B)** and viability **(C)** of sperm cells counted in caput epididymis measured in the four male groups. **(D–F)** ROS **(D)**, peroxides **(E)** and GSH **(F)** measured in sperm cells in the four male groups. Data are presented as means and SD. Bars and scattered plots are labeled with *p* values: **(A)** (*p* < 0.05 vs. F0-CON); **(B)** (*p* < 0.05 vs. F0-HFD); **(C)** (*p* < 0.05 vs. F0-CON-NAC); **(D)** (*p* < 0.05 vs. F0-HFD-NAC).

None of the groups showed changes in the liver, testis, prostate, or seminal vesicle weight and gross morphology (Table 1). Instead, NAC protected mice from epididymal fat and subcutaneous white adipose fat expansion (Table 1). Sperm viability (Figures 2A–C), reduced in the F0-HFD group, is instead preserved in the presence of NAC (Table 1). As shown by the reduced percentage of sperm cells in the caput epididymis positive to di-cholo-fluorescein (DCF-DA) (Figure 2D), and overall DCF-DA staining in sperm cells (Table 1) oxidative stress induced by the HFD (especially in caput spermatozoa) is reestablished by NAC. Similar results are observed also in the epididymal tissues where NAC re-establishes physiological redox environment as shown by ROS measured by luminol in cauda spermatozoa. None of the groups showed changes in mitochondrial redox potential (Table 1), suggesting that change in ROS content might indeed be ascribed to pro-oxidant species present in the lumen of the epididymis or derived from epididymosomes. As shown by the fluorescence of the GSH sensor TMR-IAA, the oxidative stress induced by HFD did not manifest as a reduction in GSH measured in *cauda* sperm (Table 1; Figure 2F). However, NAC increases GSH content in the NAC treated groups over the physiological levels measured in CON groups.

After 8 weeks of HFD or control diet, 13 week-old F0 males were mated with C57BL/6J inbred females. Mating in the F0 generation led to an equal number of litters for each of the two groups of fathers. After weaning, one female and one male pup from each litter were randomly selected and allocated into two new groups: the F1 generation from F0-HFD was named F1(F0-HFD) (n = 43 per sex), while the generation from F0-CON was named F1(F0-CON) (n = 43 per sex), and the F1 generation from F0 NAC was named F1(F0-HFD-NAC) and F1(F0-CON-NAC) (n = 10 per sex) (Figure 1A). Independent of their fathers, male and female offspring were fed a CON diet and did not receive NAC from weaning until sacrifice when they underwent metabolic testing, biochemical analyses, and post-mortem necropsy.

**FIGURE 3:**
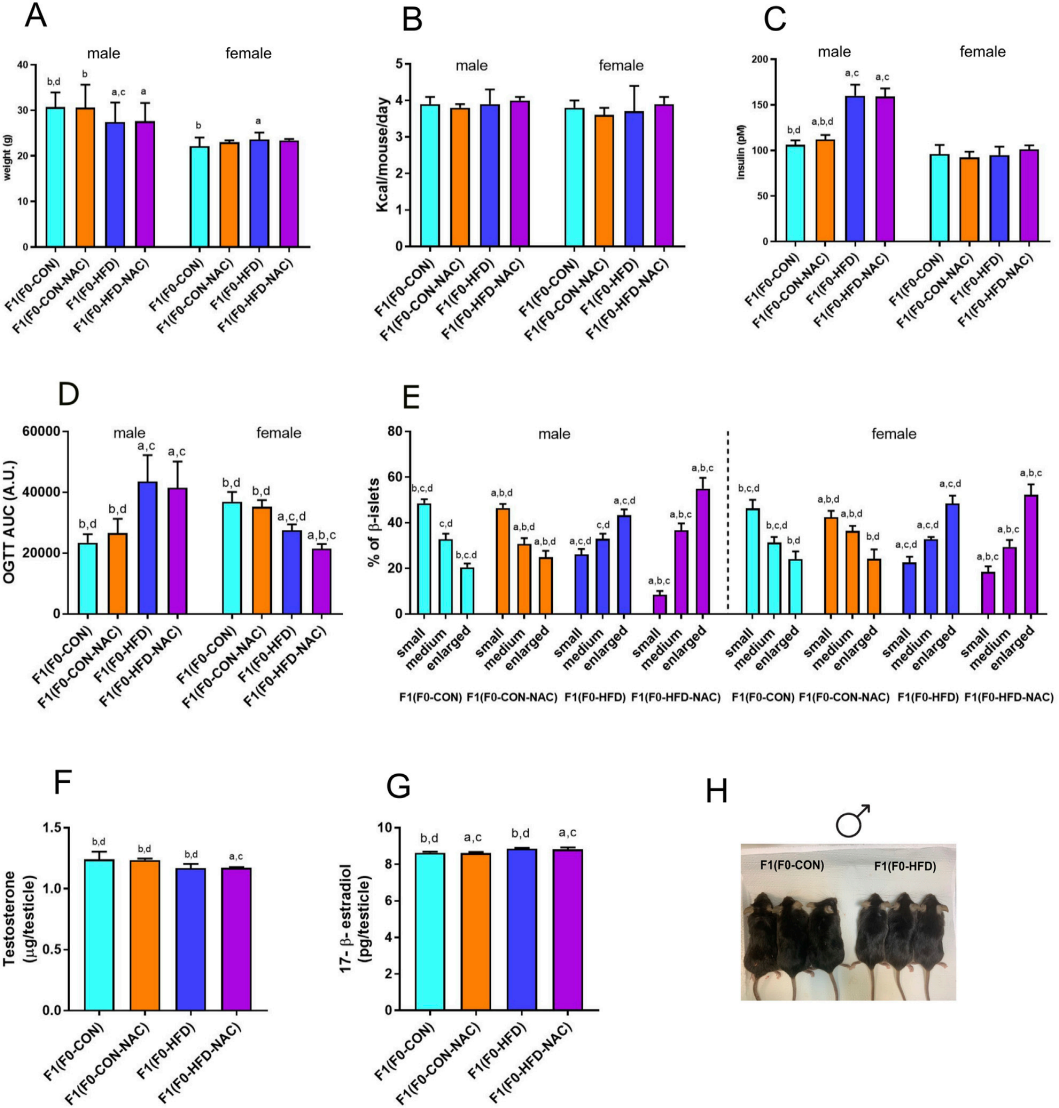
NAC does not protect F1(F0-HFD-NAC) male offspring from paternal HFD-induced predisposition to hyperinsulinemia, hyperplasia of pancreatic β-cells and insulin resistance. **(A, B)** Body weight at necropsy **(A)** and food consumption **(B)** of female and male F1(F0-CON), F1(F0-HFD), F1(F0-CON-NAC), F1(F0-HFD-NAC). **(C, D)** fasting insulinemia **(C)** and OGTT AUC **(D)** of female and male F1 offspring from different groups. **(E)** Quantification of enlarged, medium and small pancreatic islets (expressed as percentage of total number of islets) showing an increased number of large islets in pancreas of male and female F1(F0-HFD) and F1(F0-HFD-NAC). **(F, G)** Testosterone and 17-β estradiol measured in testis of the four male offspring groups. Data are presented as mean and SD. Bars are labeled with *p* values: a (*p* < 0.05 vs. F1(F0-CON)); b (*p* < 0.05 vs. F1(F0-HFD)); c (*p* < 0.05 vs. F1(F0-CON-NAC)); d (*p* < 0.05 vs. F1(F0-HFD-NAC)). **(H)** Representative picture of F1(F0-CON) and F1(F0-HFD9 males at culling.

The dysmetabolic consequences of the HFD obesogenic stimulus on F0-HFD sires on offspring sires have been previously described (Pastore et al., 2024) and are reported herein as a reference. As shown in Table 2 and Figures 3A–C, F1(F0-HFD) male pups showed reduced body weight compared to F1(F0-CON) males. NAC did not influence the weight gained by the CON or HFD group, with F1(F0-CON-NAC) and F1(F0-HFD-NAC) presenting similar weight to F1(F0-CON) and F1(F0-HFD), respectively (Figure 3A). The reduced body weight was not due to a difference in food intake, which was similar between the two groups (Table 2; Figure 3B). The leaner phenotype manifests only in the male progeny. The body weight of F1(F0-HFD) female was slightly increased compared to F1(F0-CON), suggesting a dimorphic influence of F0-HFD fathers on the progeny (Figure 3A, B).

**TABLE 2:** Morphometric (whole body and pancreas) analysis, hematochemical analysis, glycemia, insulinemia, OGTT (AUC), testosterone levels of male and female adult F1(F0-CON), F1(F0-HFD), F1(F0-CON-NAC) and F1(F0-HFD-NAC).

	*F1(F0-CON)*	*F1(F0-HFD)*	*F1(F0-CON-NAC)*	*F1(F0-HFD-NAC)*
*Male F1*	43	43	10	10
*Body Weight at sacrifice(g)*	30.7 ± 3.2^a^ ^,^ ^b^	27.4 ± 4.3^c^ ^,^ ^d^	30.6 ± 0.5^a^	27.6 ± 0.4^c^
*Food Intake (g/mice/day)*	3.9 ± 0.2	3.9 ± 0.4	3.8 ± 0.1	4.0 ± 0.1
*Clinical Biochemistry*				
*Glucose (mg/dL)*	113.4 ± 7.2^a^ ^,^ ^b^	106.0 ± 3.0^c^ ^,^ ^d^	114.6 ± 4.1^a^ ^,^ ^b^	104.8 ± 3.4^c^ ^,^ ^d^
*Insulin (pM)*	106 ± 5^a^ ^,^ ^b^	160 ± 12^c^ ^,^ ^d^	112 ± 5^a^ ^,^ ^b^	159 ± 9^c^ ^,^ ^d^
*OGTT (AUC)*	23,425 ± 2821^a^ ^,^ ^b^	43,569 ± 8,680^c^ ^,^ ^d^	26,593 ± 4,670^a^ ^,^ ^b^	41,467 ± 7,230^c^ ^,^ ^d^
*Cholesterol Total (mg/dL)*	96.3 ± 15.1	93.7 ± 11.8	95.1 ± 10.6	94.3 ± 16.1
*Triglycerides (mg/dL)*	150.6 ± 17.3	144.9 ± 18.3	145.7 ± 7.7	141.6 ± 10.9
*HDL (mg/dL)*	45.6 ± 5.2	43.7 ± 7.8	46.5 ± 6.1	41.4 ± 6.2
*LDL (mg/dL)*	9.3 ± 1.2	8.7 ± 1.3	9.9 ± 1.4	10.9 ± 1.3
*ALT (U/L)*	34.2 ± 1.8	32.5 ± 2.3	34.3 ± 1.8	33.3 ± 1.4
*AST (U/L)*	50.6 ± 3.2	52.2 ± 4.1	51.5 ± 4.5	53.5 ± 5.4
*Testosterone (ug/testicle)*	1.242 ± 0.062^a^ ^,^ ^b^	1.169 ± 0.035^c^ ^,^ ^d^	1.236 ± 0.012^a^ ^,^ ^b^	1.172 ± 0.006^c^ ^,^ ^d^
*17-β estradiol (pg/testicle)*	8.639 ± 0.053^a^ ^,^ ^b^	8.860 ± 0.054^c^ ^,^ ^d^	8.621 ± 0.062^a^ ^,^ ^b^	8.823 ± 0.104^c^ ^,^ ^d^
*Pancreatic β-islets Pancreatic islets*				
*% Small (0–5,000* * * *μm* ^*2*^ *)*	48.5 ± 1.8^a^ ^,^ ^d^ ^,^ ^b^	26.1 ± 2.4^c^ ^,^ ^d^ ^,^ ^b^	46.4 ± 1.8^c^ ^,^ ^a^ ^,^ ^b^	8.4 ± 1.7^c^ ^,^ ^a^ ^,^ ^d^
*% Medium (5,001–10000* * * *μm* ^*2*^ *)*	32.8 ± 2.4^d^ ^,^ ^b^	32.9 ± 2.3^d^ ^,^ ^b^	30.7 ± 2.5^c^ ^,^ ^a^ ^,^ ^b^	36.7 ± 3.0^c^ ^,^ ^a^ ^,^ ^d^
*% Large (>10,000* * * *μm* ^*2*^ *)*	20.4 ± 1.7^a^ ^,^ ^d^ ^,^ ^b^	43.2 ± 2.6^c^ ^,^ ^d^ ^,^ ^b^	24.9 ± 2.8^a^ ^a^ ^,^ ^b^	54.9 ± 4.8^c^ ^,^ ^a^ ^,^ ^d^
*Female F1*	43	43	10	10
*Body Weight at sacrifice(g)*	22.1 ± 1.9^a^	23.6 ± 1.5^c^	23.0 ± 0.4	23.4 ± 0.3
*Food Intake (g/mice/day)*	3.8 ± 0.2	3.7 ± 0.7	3.6 ± 0.2	3.9 ± 0.2
*Clinical Biochemistry*				
*Glucose (mg/dL)*	102.0 ± 4.3^a^ ^,^ ^b^	123.0 ± 7.4^c^ ^,^ ^d^	105.6 ± 4.4^a^ ^,^ ^b^	120.5 ± 2.3^c^ ^,^ ^d^
*Insulin*	96.0 ± 10.0	95.1 ± 9.2	92.4 ± 6.2	101.3 ± 4.2
*OGTT (AUC)*	36,873 ± 3,253^a^ ^,^ ^b^	27,546 ± 1952^c^ ^,^ ^d^ ^,^ ^b^	35,225 ± 2,216^a^ ^,^ ^b^	21,473 ± 1,519^c^ ^,^ ^a^ ^,^ ^d^
*Cholesterol Total (mg/dL)*	92.0 ± 11.2	90.4 ± 9.3	90.2 ± 8.8	91.2 ± 10.3
*Triglycerides (mg/dL)*	122.7 ± 10.1	124.9 ± 11.4	119.3 ± 5.7	129.3 ± 7.7
*HDL (mg/dL)*	42.6 ± 3.5	41.6 ± 4.9	44.7 ± 4.6	39.6 ± 4.4
*LDL (mg/dL)*	11.4 ± 2.2	10.9 ± 1.9	11.5 ± 1.6	12.0 ± 1.1
*ALT (U/L)*	32.4 ± 1.1	30.6 ± 1.7	32.4 ± 1.3	28.8 ± 0.9
*AST (U/L)*	51.7 ± 2.3	50.2 ± 3.7	53.8 ± 5.0	50.7 ± 4.9
*Pancreatic β-islets Pancreatic islets*				
*% Small (0–5,000* * * *μm* ^*2*^ *)*	46.2 ± 3.8^a^ ^,^ ^d^ ^,^ ^b^	22.6 ± 2.5^c^ ^,^ ^d^ ^,^ ^b^	42.4 ± 2.8^c^ ^,^ ^a^ ^,^ ^b^	18.4 ± 2.5^c^ ^,^ ^a^ ^,^ ^d^
*% Medium (5,001–10000* * * *μm* ^*2*^ *)*	31.3 ± 2.5^a^ ^,^ ^d^ ^,^ ^b^	32.8 ± 1.0^c^ ^,^ ^d^ ^,^ ^b^	36.4 ± 2.2^c^ ^,^ ^a^ ^,^ ^b^	29.4 ± 3.0^c^ ^,^ ^a^ ^,^ ^d^
*% Large (>10,000* * * *μm* ^*2*^ *)*	24.1 ± 3.3^a^ ^,^ ^b^	48.4 ± 3.5^c^ ^,^ ^d^ ^,^ ^b^	24.2 ± 4.1^a^ ^,^ ^b^	52.2 ± 4.6^c^ ^,^ ^a^ ^,^ ^d^

Results are reported as mean ± SD., Statistically significant difference and values *p* values <0.05 are indicated with letters.

^a^ (*p* < 0.05 vs. F1(F0-HFD)).

^b^ (*p* < 0.05 vs. F1(F0-HFD-NAC)).

^c^ (*p* < 0.05 vs. F1(F0-CON)).

^d^ (*p* < 0.05 vs. F1(F0-CON-NAC)).

NAC did not protect the offspring from paternal intergenerational dysmetabolism. Despite fed control diet and independently from NAC, both F1(F0-HFD) and F1(F0-HFD-NAC) male presented a severe diabetic phenotype with fasting hyperinsulinemia and glucose resistance as shown by the OGTT (Table 2; Figures 3C, D). As for weight gain, the diabetic phenotype was sexually dimorphic, with F1(F0-HFD) female presenting fasting insulin levels not different from F1(F0-CON) group (Table 2; Figures 3C, D), and, intriguingly, augmented performance in the OGTT. We have already shown that fasting hyperinsulinemia in F1(F0-HFD) male mice results from pancreatic β−cell expansion (Rieck and Kaestner, 2010; Pastore et al., 2024). In addition, paternal NAC did not correct the altered phenotype of the offspring. Indeed, histological analysis of pancreatic tissues of F1(F0-HFD) confirmed an increased percentage of enlarged pancreatic islets (Table 2; Figure 3E). Intriguingly, pancreatic β-cell expansion occurs also in F1(F0-HFD) female, where we as well measured an increased percentage of enlarged pancreatic islets. Despite the diabetic phenotype and independence from NAC, liver functionality, as shown by circulating Triglycerides, HDL, LDL, ALT, and AST activities, as well as gross changes in testicular, prostate, and seminal vesicle weight and morphology, were not significantly different among the groups.

We have already shown that the dimorphic phenotype promoted by a paternal F0-HFD depends on alterations in testosterone and 17-β estradiol levels (Pastore et al., 2024). To verify whether paternal NAC was able to avoid alteration of testosterone and 17-β estradiol levels in the male progeny of F1(F0-HFD) males, we measured the testicular levels of the two hormones in the male offspring of the four groups. NAC was unable to prevent testosterone reduction in the male progeny of F0-HFD-NAC. Indeed, F1(F0-HFD-NAC) present testosterone (F1(F0-HFD): 1.169 ± 0.035; F1(F0-CON): 1.242 ± 0.062; F1(F0-CON-NAC): 1.236 ± 0.012; F1(F0-HFD-NAC): 1.172 ± 0.006 g)) and 17-β estradiol levels (F1(F0-HFD): 8.860 ± 0.054; F1(F0-CON): 8.639 ± 0.053; F1(F0-CON-NAC): 8.621 ± 0.062; F1(F0-HFD-NAC): 8.823 ± 0.104 g)) similar to F1(F0-HFD) males ((F1(F0-HFD) (Table 2; Figures 3F, G).

### 3.3 NAC does not protect male offspring from HFD-induced epigenetic alterations occurring at IgfII-H19 ICR and cyp19a1 exon I loci

**FIGURE 4:**
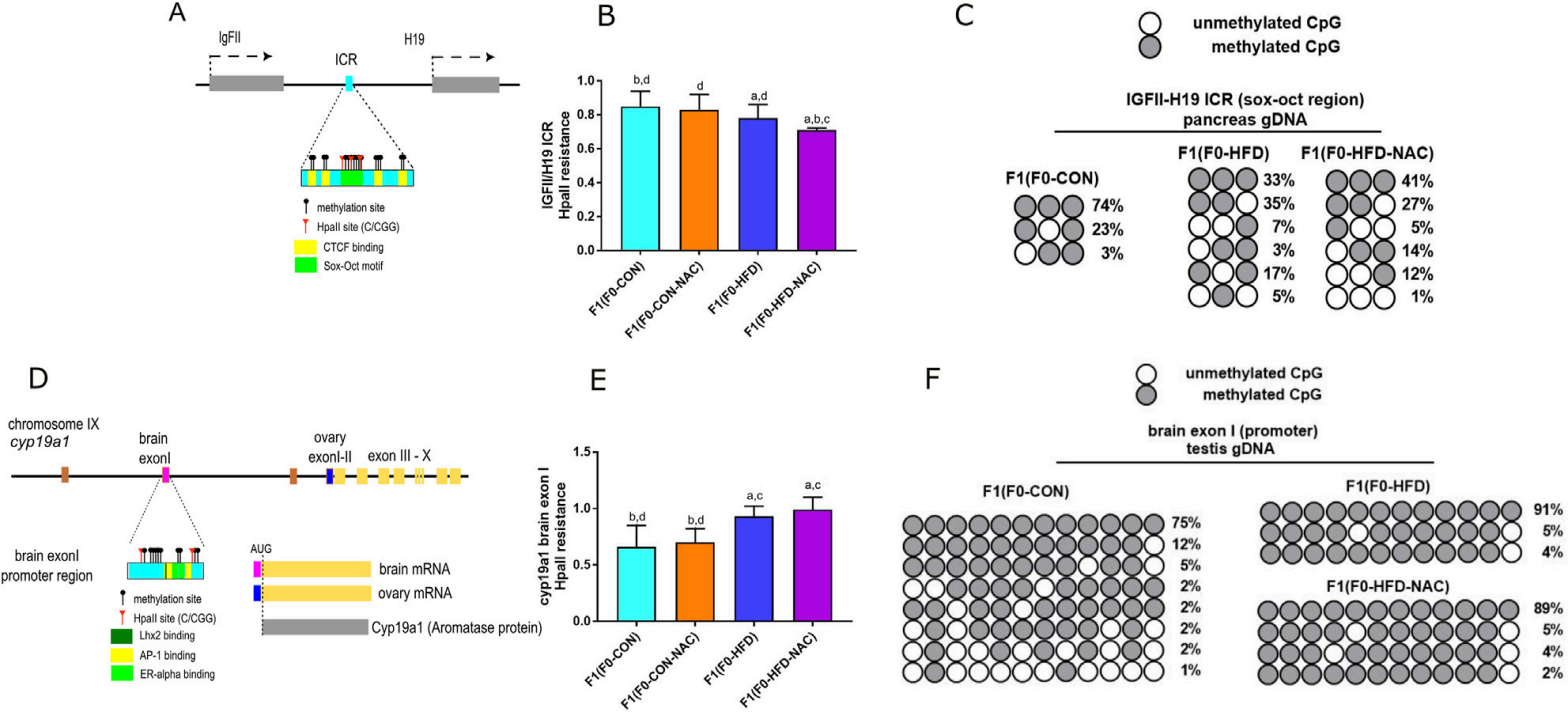
Independently from NAC, F0-HFD sires predispose male offspring to altered methylation pattern at the *IGF-II* and *H19* enhancer region and *cy9a1* exon I promoter region. **(A)** Schematic representation of *IGF-II/H19* enhancer region on mouse chromosome VII. CpG cytosines, HpaII (C/CGG) restriction sites, CTCF biding sites (yellow) and the Sox-Oct motif (light green) are indicated; **(B)** Methylation sensitive qPCR analysis of gDNA extracted from pancreas of male F1(F0-HFD), F1(F0-CON), F1(F0-HFD-NAC), F1(F0-CON-NAC) revealing significant reduction in methylation levels of pancreatic *IGF-II* and *H19* ICR region in F1(F0-HFD) and F1(F0-HFD-NAC) (For analysis in A, fold change is expressed as 2^−ΔΔCT^ vs. undigested gDNA). **(C)** Sanger sequencing of bisulfite converted pancreatic gDNA of F1(F0-HFD), F1(F0-CON), F1(F0-HFD-NAC) groups. The analysis confirmed that, independently from NAC, CpGs allocated in one of the subregions of the SOX/OCT binding site of the ICR in F1(F0-HFD) and F1(F0-HFD-NAC) males present a reduced pattern of methylation compared to F1(F0-CON) males. **(D)** Schematic representation of *cyp19a1* gene locus on chromosome IX. CpG cytosines, HpaII restriction sites, Oestrogen Receptor (pale yellow), Jun/Fos (AP1 light green) and Lhx2 binding sites (dark green) are indicated; **(E)** Methylation sensitive qPCR analysis of gDNA extracted from testis of F1(F0-HFD), F1(F0-CON), F1(F0-HFD-NAC), F1(F0-CON-NAC) revealing significant increase in methylation levels in *cyp19a1* brain exon I region in F1(F0-HFD) and F1(F0-HFD-NAC) (For analysis in B, fold change is expressed as 2^−ΔΔCT^ vs. undigested gDNA). **(F)** Sanger sequencing of bisulphite converted testis gDNA of F1(F0-HFD) and F1(F0-CON). The analysis confirmed increased methylation of cytosines in *cyp19a1*brain exon I of F1(F0-HFD) and F1(F0-HFD-NAC) testis compared to F1(F0-CON). Data are presented as mean and SD. Bars are labeled with *p* values: **(A)** (*p* < 0.05 vs. F1(F0-CON)); **(B)** (*p* < 0.05 vs. F1(F0-HFD)); **(C)** (*p* < 0.05 vs. F1(F0-CON-NAC)); **(D)** (*p* < 0.05 vs. F1(F0-HFD-NAC)).

Intergenerational transmission of dysmetabolic traits promoted by an HFD involves altered genomic DNA methylation during embryonic development. We have shown that as result of 8 weeks of paternal HFD, at least two genetic loci result epimutated in F1(F0-HFD) offspring (Pastore et al., 2024). The first epimutation occurs in pancreatic gDNA at the enhancer region located between the *IGF-II* and the *H19* genes on mouse chromosome VII (Figure 4A)*.* The methylation status of this enhancer can influence the selective expression of the *IGFII* gene (Ito et al., 2013), which codes for a growth factor that dictates pancreatic development at the embryonic stage (Darbandi et al., 2019a; Darbandi et al., 2019b; Sandovici et al., 2021). Methylation sensitive PCR analysis confirm that cytosines allocated in a subregion of the SOX/OCT binding site of the ICR were statistically less methylated in pancreatic cells of F1(F0-HFD) and F1(F0-HFD-NAC) males compared to F1(F0-CON) males (300 bp, - strand of Chr IX: *54,136,106–54,136,406*) (F1(F0-HFD): 0.78 ± 0.08; F1(F0-CON): 0.85 ± 0.09; F1(F0-HFD-NAC): 0.710 ± 0.02; F1(F0-CON-NAC): 0.830 ± 0.09 (2^−Δct^ vs. undigested gDNA)) (Figure 4B). As shown in Figure 4C, Sanger sequencing of the bisulfite-converted pancreatic gDNA of male F1(F0-HFD-NAC) showed hypomethylated ICRs.

The second region affected by paternal HFD is gonadic *cyp19a1* (Figure 4D), located on chromosome IX (Golovine et al., 2003; Honda et al., 1996). We have already shown that, as a consequence of paternal HFD, the brain promoter/exon I locus of *cyp19a1* exhibits increased methylation (300 bp, - strand of Chr IX: *54,136,106–54,136,406*). As shown in Figures 4E, F, NAC did not protect against paternal HFD-induced brain exon I hypermethylation. Methylation sensitive qPCR analysis reveals that methylation status at this region is similar in F1(F0-HFD) and F1(F0-HFD-NAC) testis (Figure 4E) (F1(F0-HFD): 0.93 ± 0.09; F1(F0-CON): 0.66 ± 0.19, F1(F0-HFD-NAC): 0.99 ± 0.11; F1(F0-CON-NAC): 0.70 ± 0.12 (2^−Δct^ vs. undigested gDNA). Sanger sequencing of bisulfite-converted testis gDNA of male F1(F0-HFD-NAC) confirmed the failure of NAC to protect against intergenerational inheritance of epimutations occurring at this genetic locus (Figure 4F).

## 4 Discussion

Here, we show that NAC protects mice from systemic (obesity, glucose intolerance, and insulin resistance) and gonad-specific (epididymal adiposity, epididymal, and gonadal oxidative stress) effects of an obesogenic diet, making the health status and morphometric parameters of HFD-fed fathers comparable to those of mice fed a control diet. Despite preservation of paternal health status, consumption of NAC did not hamper epigenetic transmission of dysmetabolic traits to the offspring, that, similar to those born from the NAC-untreated HFD-fed group, presented paternally imprinted hypoandrogenism, growth retardation, and diabetes.

It is nowadays accepted that the underlying mechanism of paternal intergenerational inheritance involves altered epigenetic modifications transmitted from fathers to offspring *via* sperm cells and seminal fluid (Breton et al., 2021). Altered DNA methylation patterns, histone modification/retention, and small non-coding RNAs have been included as vehicles of paternal epigenetic information in zygotes (Perez and Ben, 2019; Champroux et al., 2018). Gluckman and Hanson (Gluckman et al., 2005) hypothesized that this non-Mendelian transmission of genetic information allows parents to inform offspring of the environment (including food availability and composition) they will experience, which would most likely permit a better pre-adaptation to it. However, attempts to achieve a better biological fit are deleterious when there is a mismatch between the developmental and post-developmental environments experienced by the offspring. Many authors have hypothesized that intergenerational inheritance, when fully confirmed in humans, could represent one of the undercover contributors to the modern spreading of metabolic diseases, including type II diabetes (Lillycrop and Burdge, 2011; Hypponen et al., 2003; Horsthemke, 2018). The prevalence of metabolic diseases, such as type 2 diabetes and obesity, is increasing at an alarming rate worldwide. Although it is accepted that personal life habits and individual genetic variation alone cannot explain this increase (Phelps et al., 2024; Wang and Lobstein, 2006), evidence suggests that environmental and epigenetic factors contribute significantly to these trends (Lillycrop and Burdge, 2011; King and Skinner, 2020). Pre-conceptional parental lifestyles are emerging as probable contributing factors to the spread of dysmetabolism in children (Nilsson et al., 2018; Lindsay et al., 2000).

Despite the progress in scientific fields interested in intergenerational inheritance, many aspects remain to be elucidated. Among the others, the series of mechanistic events “transducing” paternal environmental stimuli into sperm epigenetic changes are still unknown. For the intergenerational transmission of paternal HFD-driven dysmetabolism, the most likely hypothesis is that the paternal health state might represent itself as the transducer for intergenerational inheritance. Indeed, sires receiving a HFD manifest hyperplasia and hypertrophy of visceral fat (in mice mostly surrounding the epididymis and testis), gonadal inflammation, reduction of testosterone levels, and in the most severe case, leukocyte infiltration and alteration of the blood/testicular barrier (Figueroa-Colon et al., 2000).

Obesity causes an increase in circulating ROS and systemic oxidative stress. ROS are necessary for sperm in mammals and are involved in epididymal sperm cells entrance into senescence and are necessary for sperm capacitation, hyperactivated motility of sperm flagellum and sperm binding to *zona pellucida*. ROS control DNMTs activity and determine gDNA hypomethylation, hampering the ability of DNMTs to recognize deoxy-guanosine in CpG islands.

If ROS indeed represents the transducing link connecting the health status of fathers and the epigenome of sperm cells, then antioxidants acting on the gonads of fathers would represent pharmaceutical opportunities to interrupt paternal intergenerational inheritance. Antioxidants (Vitamin E, Selenium, NAC) has been shown to reduce the amount of seminal malonaldehyde and 4-hydroxy-2 nonanal (two by-products of ROS-derived lipid peroxidation) and restore the physiological levels of ROS, ultimately improving semen quality in men with asthenozoospermia (Jannatifar et al., 2019).

Our results indicate that, at least in mice, protecting fathers from the acquisition of HFD-induced dysmetabolic systemic parameters and ROS does not protect offspring from improper epigenetic stimuli. Future experiments will be required to understand whether the HFD chemical composition, rather than its effect on sire health, might contain molecular information influencing the sperm epigenome. Similarly, recent evidence suggests that postbiotic metabolites produced by the microbiota (Korgan et al., 2022) or endocrine molecules stimulated by food consumption may act as transducers of epigenetic signatures (Hehar et al., 2017).

Finally, our results indicated that, at least for offspring phenotypes influenced by HFD and involving change in the methylation status at *IgFII-H19* and *cyp19a1* loci, gonadal ROS do not represent molecules that transduce paternal environmental stimuli into sperm and zygote epigenomes. However, this study had several limitations. First, only one dose of NAC was administered. We cannot exclude the possibility that different doses of NAC would have been sufficient to block deleterious paternal epigenetic transmission. Indeed, as shown in Figure 2 and Table 1, epididymal GSH levels of NAC-treated sires were higher than those in the testes of CON- and HFD-fed sires. It has been reported that an excessively reducing environment (very low levels of ROS and peroxide and excessive levels of GSH) exerts a negative effect on cells and tissues. This hormetic effect has been observed and described for many antioxidants and is ascribed to factors contributing to the antioxidant paradox, in which an overabundance of antioxidants can be detrimental rather than beneficial (Agarwal et al., 2016). Although some studies have suggested that NAC may improve semen quality by enhancing the body’s antioxidant defenses and reducing harmful ROS in semen, evidence regarding its effectiveness is mixed, and more research is needed to fully understand the optimal dosage, efficacy, and long-term benefits of NAC supplementation in treating male infertility (Agarwal et al., 2016; Tremellen, 2008).

Another limitation is the use of antioxidant molecules that are strongly connected to intracellular pathways leading to DNA methylation. NAC is a precursor of cysteine and glutathione (GSH). However, cysteine and homocysteine are also part of the methionine-homocysteine pathway, which is strongly involved in (SAM) production. SAM is a substrate for DNMTs activity and contributes with its methyl group to cytosine methylation of CpG islands. We cannot exclude the possibility that while removing oxidative stress in the gonads, NAC may have indirectly altered DNMTs enzymatic activities. Future experiments will be required to test whether different antioxidants, with different impacts on DNMTs activity (such as PUFA or vitamin C), might remove excess gonadal ROS and be effective in reducing intergenerational transmission.

## Data Availability

The original contributions presented in the study are included in the article/supplementary material, further inquiries can be directed to the corresponding author.
